# Geographical distribution of American cutaneous leishmaniasis and its phlebotomine vectors (Diptera: Psychodidae) in the state of São Paulo, Brazil

**DOI:** 10.1186/1756-3305-3-121

**Published:** 2010-12-20

**Authors:** Paloma Helena Fernandes Shimabukuro, Túllio Romão Ribeiro da Silva, Frederico Octávio Fonseca Ribeiro, Luke Anthony Baton, Eunice Aparecida Bianchi Galati

**Affiliations:** 1 Instituto Leônidas e Maria Deane, FIOCRUZ - Amazônia, Rua Terezina, 476, Adrianópolis, Manaus, Amazonas, Brazil; 2 Universidade Estadual do Amazonas, Avenda Djalma Batista, 2470, Chapada, Manaus, Amazonas, Brazil; 3 Instituto Nacional de Pesquisa da Amazônia, Avenida André Araújo, 2936, Aleixo, Manaus, Amazonas, Brazil; 4 50 Rowntree Way, Saffron Walden, Essex, CB11 4DL, UK; 5 Faculdade de Saúde Pública da Universidade de São Paulo, Departamento de Epidemiologia, Avenida Dr. Arnaldo, 755, Cerqueira César, São Paulo, São Paulo, Brazil

## Abstract

**Background:**

American cutaneous leishmaniasis (ACL) is a re-emerging disease in the state of São Paulo, Brazil. It is important to understand both the vector and disease distribution to help design control strategies. As an initial step in applying geographic information systems (GIS) and remote sensing (RS) tools to map disease-risk, the objectives of the present work were to: (i) produce a single database of species distributions of the sand fly vectors in the state of São Paulo, (ii) create combined distributional maps of both the incidence of ACL and its sand fly vectors, and (iii) thereby provide individual municipalities with a source of reference material for work carried out in their area.

**Results:**

A database containing 910 individual records of sand fly occurrence in the state of São Paulo, from 37 different sources, was compiled. These records date from between 1943 to 2009, and describe the presence of at least one of the six incriminated or suspected sand fly vector species in 183/645 (28.4%) municipalities. For the remaining 462 (71.6%) municipalities, we were unable to locate records of any of the six incriminated or suspected sand fly vector species (*Nyssomyia intermedia*, *N. neivai*, *N. whitmani*, *Pintomyia fischeri*, *P. pessoai *and *Migonemyia migonei*). The distribution of each of the six incriminated or suspected vector species of ACL in the state of São Paulo were individually mapped and overlaid on the incidence of ACL for the period 1993 to 1995 and 1998 to 2007. Overall, the maps reveal that the six sand fly vector species analyzed have unique and heterogeneous, although often overlapping, distributions. Several sand fly species - *Nyssomyia intermedia *and *N. neivai *- are highly localized, while the other sand fly species - *N. whitmani, M. migonei, P. fischeri *and *P. pessoai *- are much more broadly distributed. ACL has been reported in 160/183 (87.4%) of the municipalities with records for at least one of the six incriminated or suspected sand fly vector species, while there are no records of any of these sand fly species in 318/478 (66.5%) municipalities with ACL.

**Conclusions:**

The maps produced in this work provide basic data on the distribution of the six incriminated or suspected sand fly vectors of ACL in the state of São Paulo, and highlight the complex and geographically heterogeneous pattern of ACL transmission in the region. Further studies are required to clarify the role of each of the six suspected sand fly vector species in different regions of the state of São Paulo, especially in the majority of municipalities where ACL is present but sand fly vectors have not yet been identified.

## Background

Phlebotomine sand flies are dipteran insects and some species are vectors of *Leishmania *spp., the causative agents of American cutaneous leishmaniasis (ACL). The disease has been known to occur in the state of São Paulo at least since 1884 [1]. American cutaneous leishmaniasis transmission in São Paulo state initially increased as a consequence of deforestation during the expansion of coffee plantations in the 19^th ^century, and then continued, in the first decades of the 20^th ^century, as coffee farms became widespread towards the northwestern area of the state and railway roads were built to transport coffee grains to the coast [1]. After declining in incidence by the end of the 1950's, ACL became endemic in southern areas of the state in the 1970's [1]. In the 1980's, ACL was considered a re-emergent disease due to anthropic factors and was not directly related to forested environments, as it had been historically [2]. This represented a new feature of transmission, marked by micro-outbreaks or isolated cases scattered throughout the state in rural or peri-urban areas [2,3]. Transmission of the *Leishmania *spp. causing ACL in these areas depends on the adaptation of potential vectors species to anthropic environments and involves domestic animals [4,5].

In the state of São Paulo, the main etiological agent of ACL is *Leishmania *(*Viannia*) *braziliensis *[6]. Five sand fly species are incriminated vectors of *L*. (*V*.) *braziliensis*: *Nyssomyia intermedia, Nyssomyia neivai, Nyssomyia whitmani, Migonemyia migonei *and *Pintomyia pessoai *[2,7,8]; while one other species is a suspected vector: *Pintomyia fischeri *[2]. *Leishmania *(*Leishmania*) *forattinii *[9] and *Leishmania *(*Leishmania*) *amazonensis *[6] have also been isolated from wild reservoirs, and a small number of human ACL cases caused by *L*. (*L*.) *amazonensis *have been reported in the north-eastern region of São Paulo State [10]. However, the only known vector of *L*. (*L*.) *amazonensis*, the sand fly *Bichromomyia flaviscutellata*, has only been recorded in a restricted area of the south coast of São Paulo state [11,12].

The complexity of ACL transmission is due to the different possible clinical outcomes (cutaneous, mucosal or diffuse) [4], the diversity of sand fly vector species and the diversity of *Leishmania *species; it is known that different sand fly species can transmit the same or a different *Leishmania *species [13]. Because vector populations are affected by humidity, temperature, vegetation, light availability and altitude [14,15], it is frequently not possible to identify the vector species in a transmission area. Also, in Brazil, it is common for the health services to report the disease based on histopathological diagnosis accompanied by Montenegro skin test. Parasite characterization is rare, so information on which *Leishmania *species is causing the disease in most areas of Brazil is unknown.

There has been a significant increase in the use of geoprocessing techniques to understand the transmission of infectious diseases. These techniques allow the analysis of data over large territorial scales and the production of disease maps, ecological analyses, prediction of parasite occurrence and surveillance for various parasitic, including arthropod-borne, diseases [16,17]. Regarding leishmaniasis, geographic information systems (GIS) and remote sensing (RS) techniques have been used to: (i) map disease occurrence, and (ii) build predictive models [18]. With respect to phlebotomine sand flies in the state of São Paulo, GIS/RS techniques have been used for ecological niche modelling of vector species of ACL [18,19], and to map disease and/or vector occurrence [2,20]. However, this previous work used data on vector distribution or disease occurrence from limited time periods.

In the present work, we gathered data on the distribution of sand flies in the state of São Paulo during the past 65 years (the period from 1943 to 2009), and compared this data to the distribution of notified ACL cases during the periods from 1993 to 1995 and 1998 to 2007. The aims of the present work were to: (i) collect and then organize the distribution records for sand fly species in the state of São Paulo into a single database, (ii) produce distributional maps of the phlebotomine vectors of ACL and superimpose these data with ACL disease records for the past 15 years, and (iii) thereby provide individual municipalities with a source of reference material for work carried out in their area.

## Results

In order to create maps comparing the distributions of ACL and the six incriminated or suspected sand fly vectors of *L*. (*V*.) *braziliensis*, a single database was constructed combining data on the incidence of ACL and the occurrence of the six different sand fly species investigated, for each of the 645 municipalities of the state of São Paulo (the database is available on request from PHFS). Data on ACL was obtained from the National Database on Reportable Diseases/Epidemiological Surveillance Centre (SINAN/CVE) website [21], while information on the distribution of sand flies was collated from four different sources (as described in detail in the Materials and Methods).

**Figure 1:**
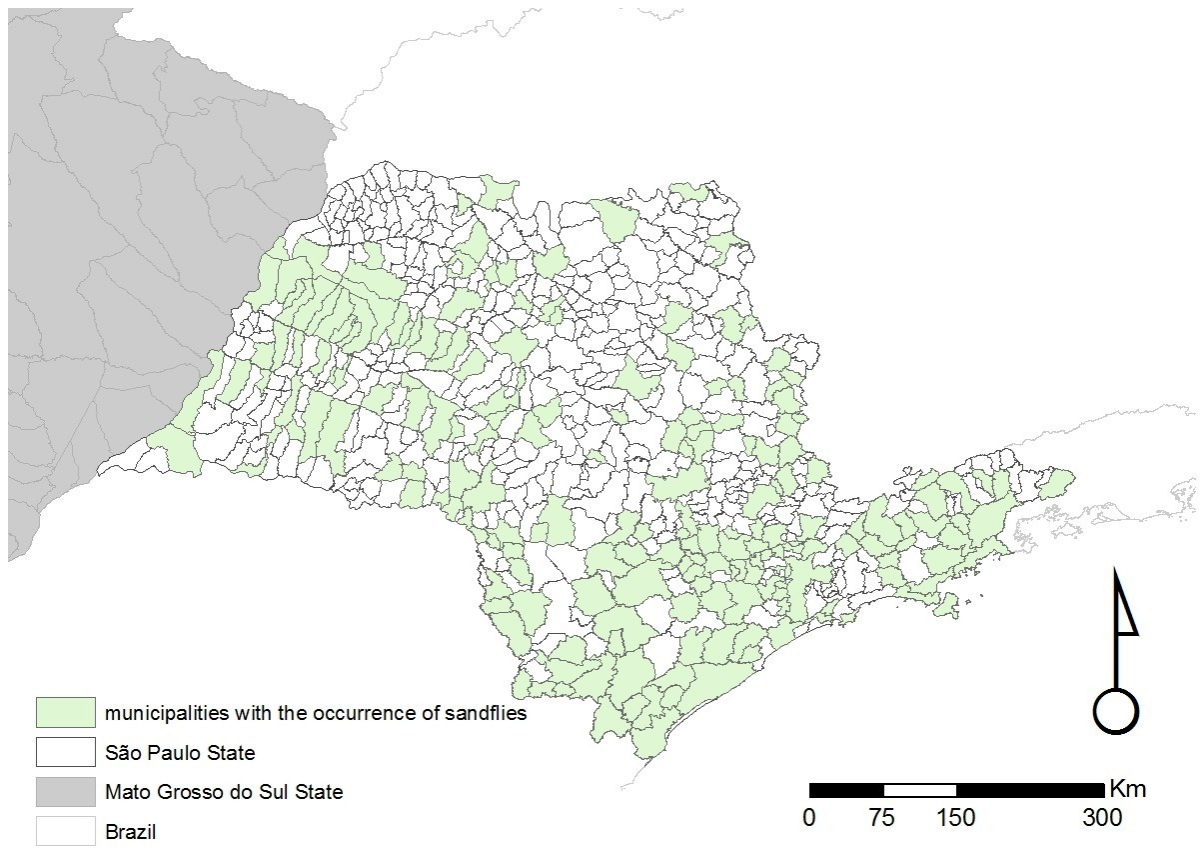
**Overall distribution of all incriminated/suspected vectors of ACL as well as all non-vector sand flies species recorded in the state of São Paulo**. Distribution of sand flies recorded in the state of São Paulo from 1943 to 2009.

**Figure 2:**
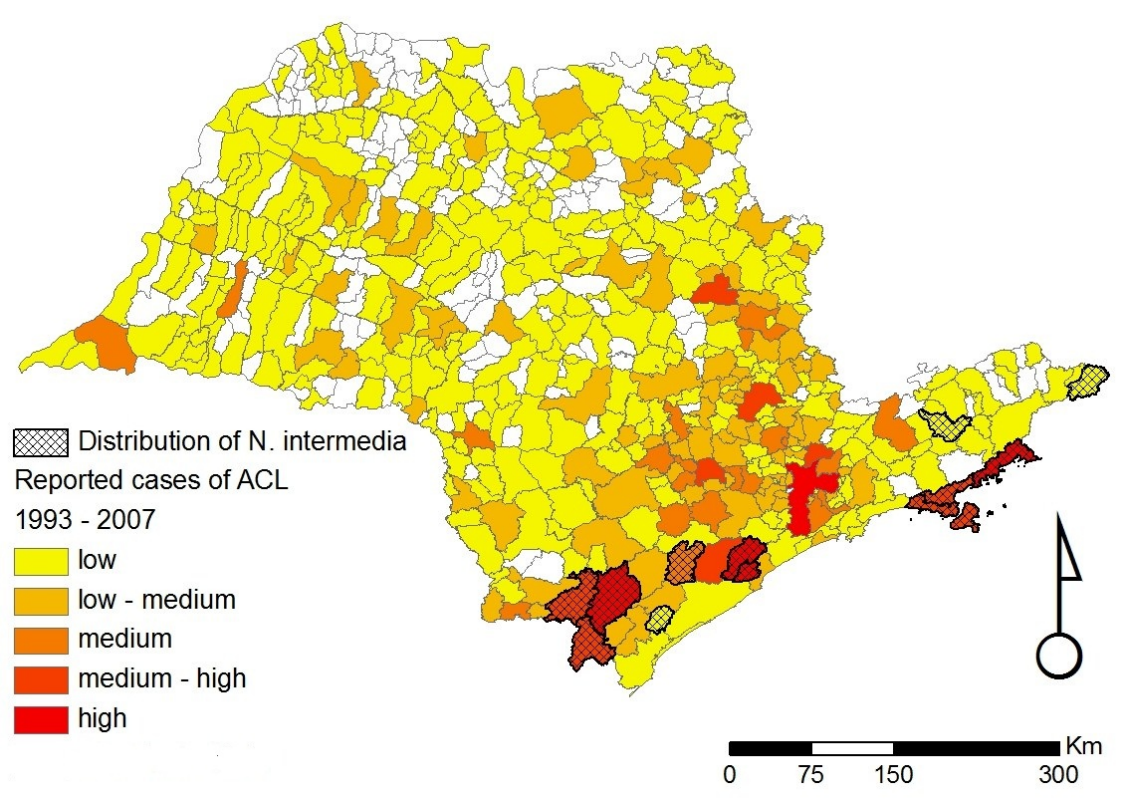
**Distribution of *Nyssomyia intermedia *in the state of São Paulo**. Distribution of *Nyssomyia intermedia *for the state of São Paulo overlaid on the incidence of ACL for the period of 1998 to 2007. The scale describing the incidence of ACL is as follows: low (1 to 10 cases), low-medium (10 to 29 cases), medium (30 to 79 cases), medium-high (80 to 147 cases), and high (148 to 468 cases).

**Figure 3:**
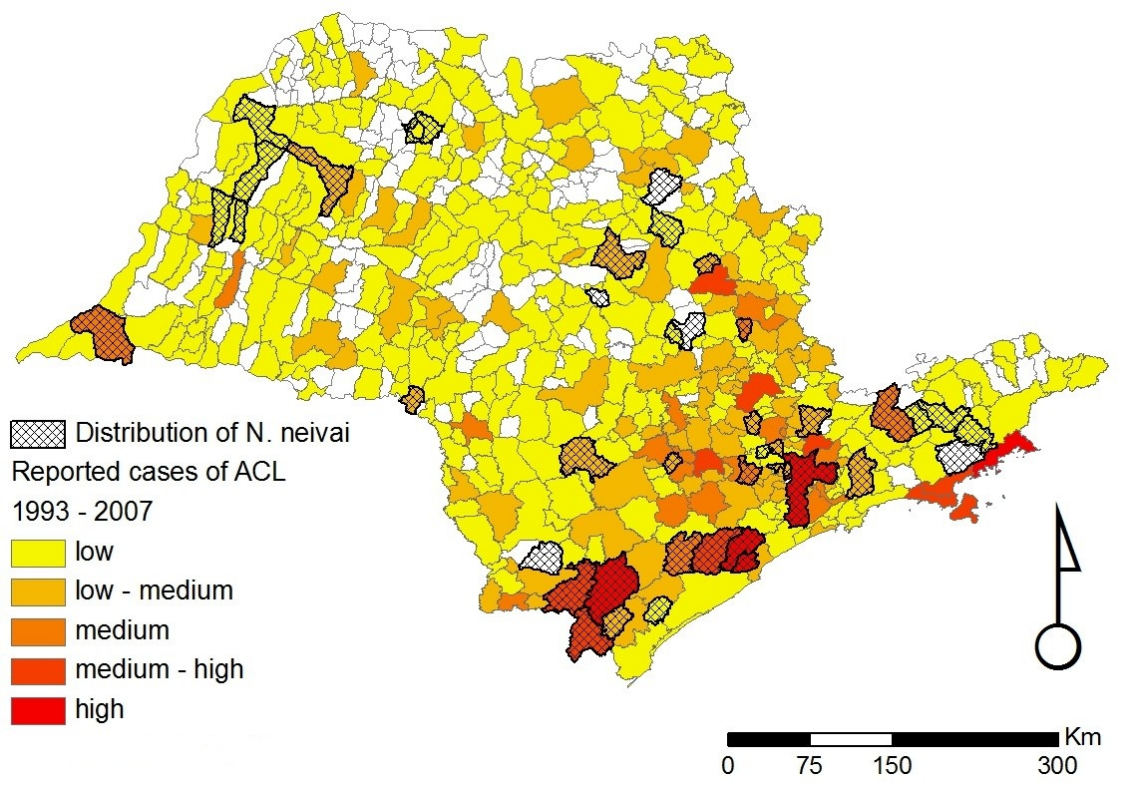
**Distribution of *Nyssomyia neivai *in the state of São Paulo**. Distribution of *Nyssomyia neivai *for the state of São Paulo overlaid on the incidence of ACL for the period of 1998- to 2007. The scale for the incidence of ACL is the same as that given in the legend of Figure 2.

**Figure 4:**
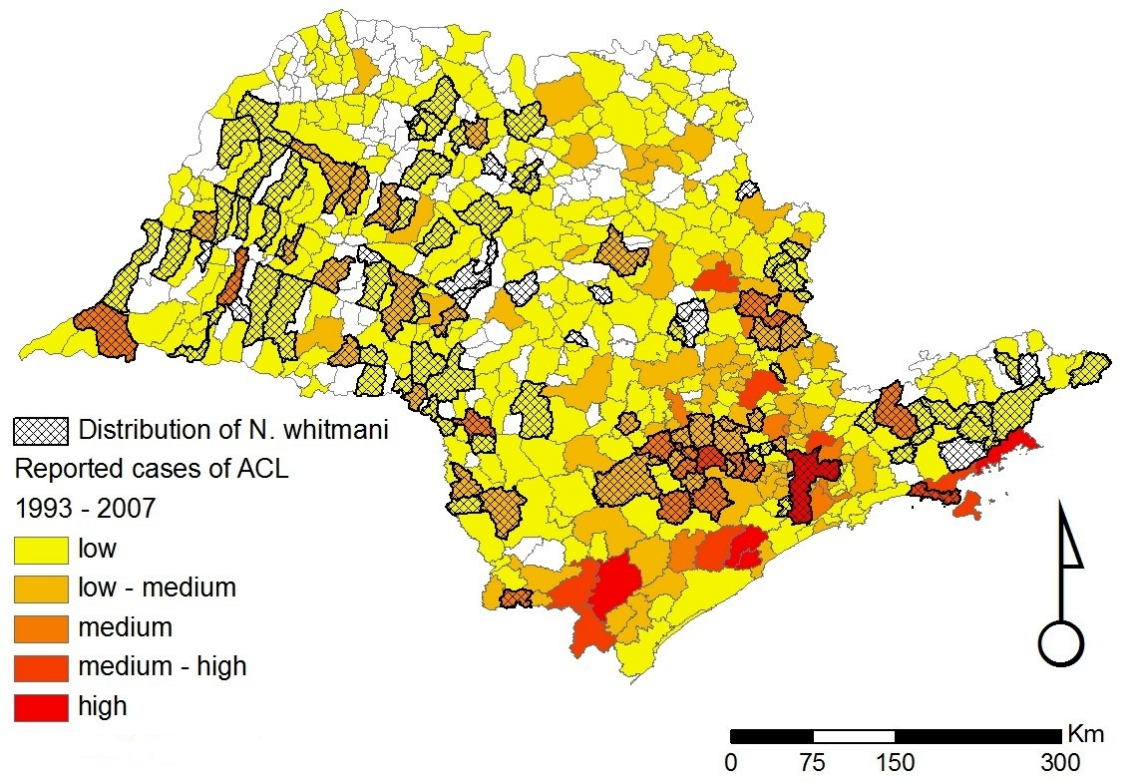
**Distribution of *Nyssomyia whitmani *in the state of São Paulo**. Distribution of *Nyssomyia whitmani *for the state of São Paulo overlaid on the incidence of ACL for the period of 1998 to 2007. The scale for the incidence of ACL is the same as that given in the legend of Figure 2.

**Figure 5:**
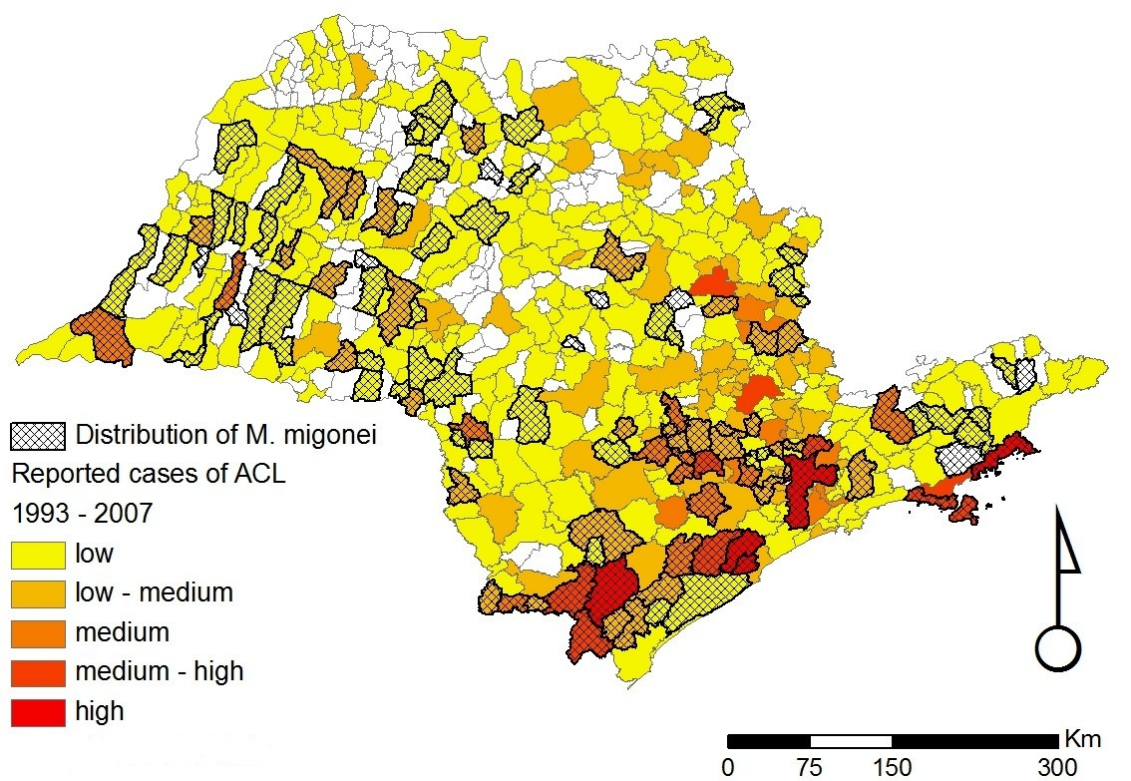
**Distribution of *Migonemyia migonei *in the state of São Paulo**. Distribution of *Migonemyia migonei *in the state of São Paulo overlaid on the incidence of ACL for the period of 1998 to 2007. The scale for the incidence of ACL is the same as that given in the legend of Figure 2.

**Figure 6:**
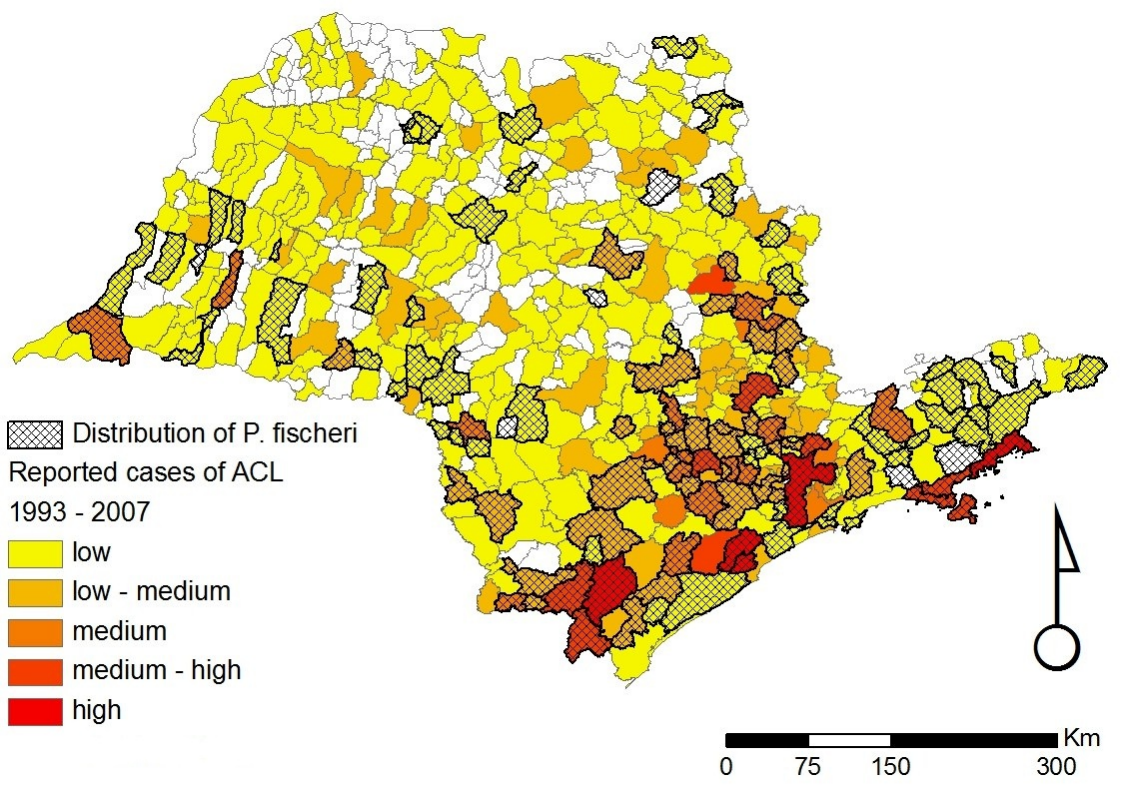
**Distribution of *Pintomyia fischeri *in the state of São Paulo**. Distribution of *Pintomyia fischeri *in the state of São Paulo overlaid on the incidence of ACL for the period of 1998 to 2007. The scale for the incidence of ACL is the same as that given in the legend of Figure 2.

**Figure 7:**
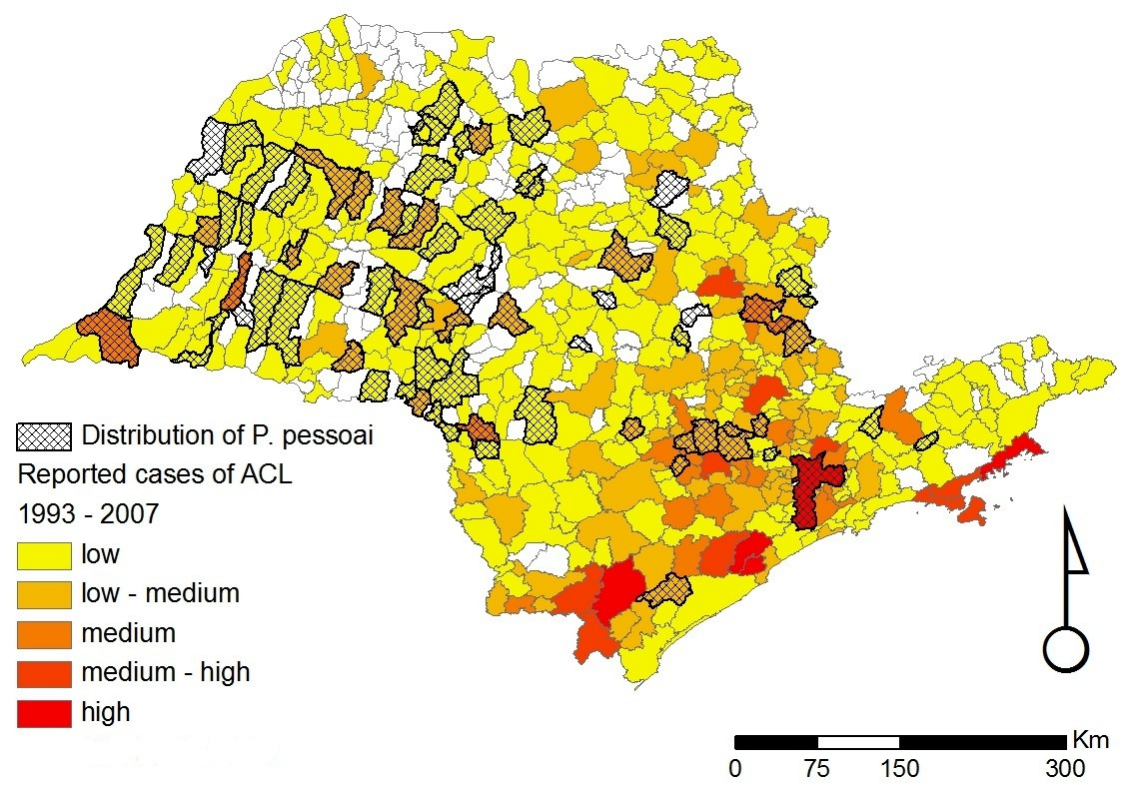
**Distribution of *Pintomyia pessoai *in the state of São Paulo**. Distribution of *Pintomyia pessoai *in the state of São Paulo overlaid on the incidence of ACL for the period of 1998 to 2007. The scale for the incidence of ACL is the same as that given in the legend of Figure 2.

Overall, our database currently contains 910 individual records, from 37 different sources, reporting the occurrence of sand flies in the state of São Paulo (Figure 1). These records date from between 1943 to 2009, and describe the presence of at least one of the six incriminated or suspected sand fly vector species in 183/645 (28.4%) municipalities, only 23 of which have no records of ACL (Figures 2, 3, 4, 5, 6 and 7). For the remaining 462 (71.6%) municipalities, we were unable to locate records of any of the six incriminated or suspected sand fly vector species. In these latter municipalities, these sand fly species have either been sought but not observed, or appropriate field collections have not been undertaken/publically reported. ACL has been reported in 318/462 (68.8%) municipalities for which there are no records of the occurrence of any of the six incriminated or suspected sand fly vector species. Most of these municipalities are located in the central and northern regions of the state. In contrast, cases of ACL have been reported in most of the municipalities (160/183; 87.4%) with records of the occurrence of at least one of the six incriminated or suspected sand fly vector species (Table 1).

**Table 1:** Number of records for the six incriminated or suspected sand fly vector species involved in ACL transmission, and the number of municipalities with records for ACL.

Vector Species	Number of municipalities	Number of municipalities with notified ACL cases	Percentage of municipalities with both vector and ACL
***Nyssomyia intermedia *s.s**.	13	13	100%

***N. neivai***	42	39	93%

***N. whitmani***	113	96	85%

***Migonemyia migonei***	116	104	90%

***Pintomyia fischeri***	124	113	91%

***P. pessoai***	79	62	78%

Number of records for the most important species involved in ACL transmission and number of municipalities with records for ACL.

Figures 2, 3, 4, 5, 6 and 7 show separately the distribution of each of the six incriminated or suspected vector species of ACL in the state of São Paulo, overlaid on the incidence of ACL for the period of 1993 to 1995 and 1998 to 2007. The total number of reported cases of ACL in the state of São Paulo between 1998 and 2007 was 6,643 [21]. During this period, ACL was reported in 478/645 (74.1%) municipalities (Figures 2, 3, 4, 5, 6 and 7). Although there is a widespread, low level of endemic ACL distributed throughout the entire state of São Paulo, there are also several highly localized areas with a relatively high incidence of ACL (Figures 2, 3, 4, 5, 6 and 7). These regions are primarily (i) the southern-most and south-eastern coastal regions of the state, which consist of the protected Atlantic forest areas, and (ii) the city of São Paulo and its associated surrounding conurbation (Figures 2, 3, 4, 5, 6 and 7).

In contrast to the widespread occurrence of ACL throughout the state of São Paulo, the incriminated or suspected sand fly vector species have more circumscribed and heterogeneous distributions (Figures 2, 3, 4, 5, 6 and 7). Several sand fly species - *N. intermedia *and *N. neivai *- are highly localized, with a very restricted distribution, and are primarily found in a small number of municipalities in the southern-most and south-eastern coastal regions of the state, where there is a high incidence of ACL (Figures 2 and 3). Other sand fly species - *N. whitmani, M. migonei, P. fischeri *and *P. pessoai *- are much more broadly distributed and found in a relatively large number of municipalities, throughout the state of São Paulo, with variable levels of ACL (Figures 4, 5, 6 and 7, and Table 1). Although the latter four sand fly species are broadly distributed, there is still a tendency for each species to be located in distinct regions of the state of São Paulo, with different levels of ACL, as described in further detail in the discussion below. For example, *P. fischeri *is widely distributed throughout the southern-most and south-eastern regions, where high levels of ACL are reported (Figure 6), while *P. pessoai *exhibits a converse pattern, primarily being widely distributed in the western region of the state of São Paulo, where there are low to medium levels of ACL (Figure 7). Although each sand fly species exhibits a unique distribution, there is considerable overlap in the distributions of the sand fly species, and in many municipalities (72/183, or 39.3% of those municipalities where at least one sand fly vector is found) more than one sand fly species has been reported. This pattern is particularly apparent for those municipalities reporting high levels of ACL, where 3 or more sand fly species often occur in apparent sympatry. For example, in the southern-most focus of high ACL incidence, four sand fly species - *N. intermedia, N. neivai, P. fischeri *and *M. migonei *- have been reported. Similarly, in the municipality comprising the city of São Paulo 5 of the 6 incriminated or suspected sand fly vectors of ACL are found: *N. neivai, N. whitmani, P. fischeri*, *P. pessoai *and *M. migonei*. However, the apparently high number of cases in this latter area probably results from mis-characterization of people seeking the higher quality medical services available in São Paulo city, the capital of the state, rather than the presence of appreciable levels of ACL transmission in this region.

## Discussion

Many vector-borne infectious diseases involve the same aetiological agent, but different vectors and hosts in different regions [22]. In the state of São Paulo, at least six different sand fly species are suspected or incriminated vectors of *Leishmania braziliensis*, the main aetiological agent of ACL. Here, we presented the distribution of *N. intermedia, N. neivai, N. whitmani, P. fischeri*, *P. pessoai*, and *M. migonei*, over a period of 65 years, and compared their distributions to the distribution of ACL between the years 1993 to 1995 and 1998 to 2007.

Our database and the figures generated from it have some limitations, reflecting the quality of the original data sources that were used during its compilation. The data presented here will show the areas where the most of the entomological work has been done. Those areas of the state of São Paulo that do not have any records, do not necessarily indicate that there are no sand flies or ACL transmission, only that no species identifications have been published from those areas, or that disease notification might have been made in other cities. Caution is also needed as some data might be inaccurate because of out-of-date and/or incorrect species identification. For example, 154 records were found for the distribution for *N. intermedia *sensu lato, which cannot be checked to confirm whether it is *N. intermedia *sensu strictu or *N. neivai *(see below). Consequently, these records were excluded from our analysis. Of these excluded records, 77.9% were from municipalities in the Atlantic and Eastern Plateau, where *N. intermedia *s. s. has not otherwise been reported (Figure 2), suggesting that these records may represent *N. neivai *and that the latter is the most widespread vector species in these areas. The remaining excluded records were from (i) apparently sympatric areas, such as the Ribeira and Paraíba rivers valleys [8], where both species are probably present (17.6% of the excluded records), and (ii) coastal regions (4.5% of the excluded records), where probably only *N. intermedia *s. s. is present, as there are no independent records of *N. neivai *in these areas. One further limitation of the present study is that we used point data to construct our database and to produce the distributional maps for each vector species, since information from the literature is generally insufficient to map species abundance (i.e. density versus absence/presence).

Patterns of ACL transmission in the state of São Paulo have been changing due to human impact on the environment. Human factors contributing to altering epidemiological patterns and the re-emergence of vector-borne diseases include changes in land use which cause redistribution of synanthropic fauna [23], urbanization, migration and population mobility, and the construction of dams, pipelines and highways that affect the landscape [23,24]. Such factors have led to changing distributions of the sand fly vectors if ACL in the state of São Paulo over the last 100 years. Below is given the description of the distribution of the six incriminated or suspected vectors of ACL in the state of São Paulo.

**Figure 8:**
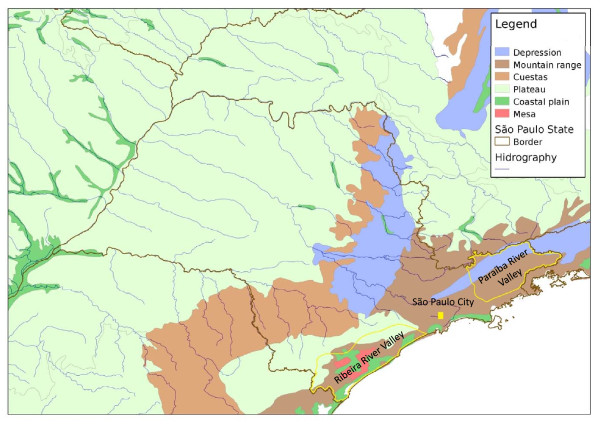
**Topography of the state of São Paulo**. Topography of the state of São Paulo including reliefs and hydrography.

*N. intermedia *was one of the first species described in the New World and also one of the first to be associated with ACL transmission in the southeast of Brazil. Today, it is known to be a complex of at least two species: *N. intermedia *s. s. and *N. neivai *[7,8]. In the state of São Paulo, *N. intermedia *s. s. is more commonly associated with lowland coastal areas, with hot and wet climates (Figures 8 and 2), where ACL transmission has increased since 2002 [25], while *N. neivai *is found at higher altitudes, with dryer climates, characteristic of the Plateau area in the west of the state (Figures 8 and 3). Because of the widespread distribution in the Plateau areas, *N. neivai *might account for most of the records for *N. intermedia *made in the past (see above). However, the two species have been found in sympatry in both southern areas (the Ribeira river valley), where ACL incidence is the highest in the state [26], as well the eastern area of the state, where it is crossed by the Mogi-Guaçu and Paraíba rivers. The latter region showed the greatest increase in the incidence of ACL between 1979 and 1992, mainly along the Mogi-Guaçu river [1] (Figures 2 and 3). In the Ribeira valley region, *N. neivai *seems to be more likely than *N. intermedia *to overcome adverse conditions in open cultivated areas and adapt to anthropic changes to the environment [27]. Thus, deforestation and changes in land use could have led *N. neivai *to occupy new areas and replace existing species in the Plateau area as observed by Odorizzi & Galati [28] and Casanova et al. [29,30]. Both species have been found naturally infected by *L. braziliensis *in other Brazilian states [31,32]; in the Plateau areas of São Paulo state, *N. neivai *(named *N. intermedia *at that time) was found naturally infected by unidentified flagellates [33,34].

*Nyssomyia whitmani *was an important vector of *L. (V.) braziliensis *in São Paulo state in the first decades of the twentieth century due to its anthropophilic habit and its high density in endemic areas during the deforestation period of the Plateau (Figure 4), and the subsequent establishment of houses that are located in either the forest or adjacent cleared areas [6,35-37]. This species might coexist with *N. intermedia *s. l. (now *N. neivai*), but might be replaced by the latter in case of anthropic activities and deforestation [38,39]. Ecological niche modelling under different global climate change scenarios of ACL vectors in south-eastern Brazil suggested that only *N. whitmani *is likely to expand its distribution in southern Brazil [18]. In this perspective, it is likely that this species might expand to the Ribeira river valley where the Atlantic forest is better preserved. This species was found naturally infected with *L*. (*V*.) *braziliensis *in other Brazilian states [40], and it was found infected by unidentified flagellates in the state of São Paulo [41].

*Migonemyia migonei *has been described as a sylvatic species that might be found at lower abundance in secondary forests [35]. This species is highly anthropophilic and may be found in animal shelters; through this adaptation to domiciliary/peridomiciliary environments, *M. migonei *can thus maintain enzootic transmission from the adjacent secondary forests [35]. *Migonemyia migonei *has a widespread distribution throughout the state (Figure 5), with records in areas of high transmission and in the Eastern Plateau, where high transmission occurred in the beginning of the twentieth century [1]. This species has been found naturally infected by *L. braziliensis *in other states of Brazil [31,40] and by undetermined *Leishmania *sp. in the state of São Paulo by Pessôa & Coutinho [41]. A correlation between the presence of this sand fly species and records of ACL cases was demonstrated by Camargo-Neves et al. [2], based on the records of sand flies captured during entomological surveillance between 1986 and 1995. *Migonemyia migonei *was found in areas where the incidence of ACL was higher, suggesting that this species might be important as a vector.

*Pintomyia fischeri *has never been found naturally infected by *Leishmania*, but it has been experimentally infected by this pathogen [41]. The anthropophily and high abundance of *P. fischeri *in transmission areas led Barretto & Coutinho [42] to believe that this species might act as a secondary vector. This species has a widespread distribution throughout the state, along the coast and in the more preserved areas of the Atlantic Plateau (Figure 6). It has been found frequently inside houses, providing further evidence that this species could act as a vector [43]. *Pintomyia fischeri *has also been recorded in more humid forested areas, being dominant in forested areas of São Paulo city and surrounding municipalities [[36], Galati, unpublished data]. It is noteworthy to mention that this species is not recorded along Tietê river course which is characterized by tropical climate with dry winter (Aw of the Koeppen climate classification system).

*Pintomyia pessoai *has been found in recently deforested areas and also occurs in human and domestic animal housing [35]. It was found naturally infected by unidentified flagellates [34,41] suggesting its involvement in ACL transmission in southeastern Brazil [13]. Gomes & Galati [44] suggested that *P. pessoai *could act as a secondary vector in the northwest-central part of São Paulo state due to its presence in primary forest. More recently, *P. pessoai *was the most abundant species collected in the northwestern region of the state [45]. This species is mostly found in the Plateau area, in patches of semi-deciduous tropical forest and the savannah-like (*cerradão*) vegetation, while it is rarely found in the hygrophilous, non-deciduous forest found along the coast (Figure 7).

## Conclusions

The maps produced in this work provide basic data on the distribution of the six incriminated or suspected sand fly vectors of ACL in the state of São Paulo, and highlight the complex and geographically heterogeneous pattern of ACL transmission in the region, in which more than one species of sand fly is often implicated. The database reported here will be updated continuously, as new epidemiological information on ACL incidence and distributional records of sand flies are published. Further studies investigating vector feeding habits and natural infection rates are required to clarify the role of each of the six suspected sand fly vector species in different regions of the state of São Paulo. New tools to improve surveillance of cases of infection in humans and non-human animal reservoirs (wild and domestic), as well as vector monitoring, also need to be developed. Further entomological work is required in the large number of municipalities where ACL is present but sand fly vectors have not yet been identified. In the latter regard, the information provided by the descriptive distribution maps presented here will, in future, provide a starting point to generate more sophisticated predictive maps of vector occurrence and disease-risk based on statistical pattern-matching [46].

## Methods

### Study area

The São Paulo state, Brazil, has a total area of 248,209.426 km^2^, which is comprised of 645 municipalities, with a total population of approximately 39,827,570 inhabitants [47]. The state of São Paulo is responsible for 33.9% of the gross domestic product (GDP) of Brazil [48].

São Paulo state is divided into five reliefs: Coastal Province, Atlantic Plateau, Peripheral Depression, Basaltic Cuestas and Eastern Plateau (Figure 8) [49]. Only 13.4% of original vegetation remains; of these, the Atlantic forest and savannah-like ecosystem (*cerradão*) are the most important, and cover 11.4% and 1.2% of the state surface, respectively (Figure 8) [50].

### Database

In order to understand and control the transmission of ACL, biological and epidemiological data have been collected by entomologists and health professionals throughout the different municipalities of the state of São Paulo for many decades. However, these data derive from disparate sources, employing diverse collection methods, and have not previously been assembled into a single resource for analysing and understanding patterns of vector distribution and ACL incidence. In the present work, we collated and systematically organized into a single database the disparate data already available on the distribution of sand flies in the state of São Paulo. The distribution of sand flies (*Nyssomyia intermedia*, *N. neivai*, *N. whitmani*, *Pintomyia fischeri*, *P. pessoai *and *Migonemyia migonei*) was based on: (i) examination of material deposited at reference entomological collections (Museu de Zoologia da Universidade de São Paulo, and Departamento de Epidemiologia/Faculdade de Saúde Pública/Universidade de São Paulo), (ii) scientific publications (papers, reports, theses), (iii) the Superintendência de Controle de Endemias/SES database [51], and (iv) personal communication with Dr. Claudio Casanova (SUCEN). In some instances, the names and boundaries of the municipalities have changed from those used in the original publications. Where we have been unable to find the current identity of these old municipalities, we have excluded the data from the database.

The nomenclature of the sand fly species follows Galati 2003 [52].

The data on the incidence of ACL in the state of São Paulo were obtained from, and are available at, the National Database on Reportable Diseases/Epidemiological Surveillance Centre SINAN/CVE website [21]. We used all the data from notified ACL cases from 1998-2007.

All the data were compiled in a Microsoft ACCESS 2007™ database, so that it was compatible with the software ArcGIS version 9.3 (Environmental Systems Research Institute, Inc., Redlands, California, United States), used in geoprocessing. The ACCESS 2007™ sand fly distribution database included the following categories: state, municipality, family, subtribe, genera, species and the references from which the data were obtained. This information was exported to the Databases Managing System (SGBD) POSTGRESQL. The POSTGRESQL software allows queries using SQL language to quantify and qualify the data. The queries were generated to visualize an event, such as distribution of a given species. The intervals describing the level of the incidence of ACL in Figures 2, 3, 4, 5, 6, 7 to distribute ACL data were: low (1 to 10), low-medium (10 to 29), medium (30 to 79), medium-high (80 to 147), and high (148 to 4680). From the ARCGIS 9.3 package, the ARCMAP and ARCCATALOG applications were used with tools from the ARCTOOLBOX, specific for geoprocessing. The database produced in this project will be made available on request from PHFS.

### Geodatabase

In order to produce the geodatabase, the Brazilian information database of the Laboratório de Sistemas de Informações Geográficas from Instituto Nacional de Pesquisas da Amazônia (SIGLAB/INPA) was used. These information is provided by the Instituto Brasileiro de Geografia e Estatística (IBGE) [48].

## Competing interests

The authors declare that they have no competing interests.

## Authors' contributions

PHFS conceived of the study, collected the data and drafted the manuscript. TRRS built the database. FORF elaborated the figures using GIS. LAB participated in the data analysis, helped to draft the manuscript, and revised the English. EABG participated in the data analysis and helped to draft the manuscript. All authors read and approved the final manuscript.
